# Nitrogen washout/washin, helium dilution and computed tomography in the assessment of end expiratory lung volume

**DOI:** 10.1186/cc7139

**Published:** 2008-12-01

**Authors:** Davide Chiumello, Massimo Cressoni, Monica Chierichetti, Federica Tallarini, Marco Botticelli, Virna Berto, Cristina Mietto, Luciano Gattinoni

**Affiliations:** 1 Dipartimento di Anestesia, Rianimazione (Intensiva e Subintensiva) e Terapia del Dolore, Fondazione IRCCS – "Ospedale Maggiore Policlinico Mangiagalli Regina Elena", via Francesco Sforza 35, 20122, Milano, Italy; 2 Istituto di Anestesiologia e Rianimazione, Fondazione IRCCS – "Ospedale Maggiore Policlinico Mangiagalli Regina Elena" di Milano, Italy; Università degli Studi di Milano, via Festa del Perdono 7, 20122, Milano, Italy

## Abstract

**Introduction:**

End expiratory lung volume (EELV) measurement in the clinical setting is routinely performed using the helium dilution technique. A ventilator that implements a simplified version of the nitrogen washout/washin technique is now available. We compared the EELV measured by spiral computed tomography (CT) taken as gold standard with the lung volume measured with the modified nitrogen washout/washin and with the helium dilution technique.

**Methods:**

Patients admitted to the general intensive care unit of Ospedale Maggiore Policlinico Mangiagalli Regina Elena requiring ventilatory support and, for clinical reasons, thoracic CT scanning were enrolled in this study. We performed two EELV measurements with the modified nitrogen washout/washin technique (increasing and decreasing inspired oxygen fraction (FiO_2_) by 10%), one EELV measurement with the helium dilution technique and a CT scan. All measurements were taken at 5 cmH_2_O airway pressure. Each CT scan slice was manually delineated and gas volume was computed with custom-made software.

**Results:**

Thirty patients were enrolled (age = 66 +/- 10 years, body mass index = 26 +/- 18 Kg/m^2^, male/female ratio = 21/9, partial arterial pressure of carbon dioxide (PaO_2_)/FiO_2 _= 190 +/- 71). The EELV measured with the modified nitrogen washout/washin technique showed a very good correlation (r^2 ^= 0.89) with the data computed from the CT with a bias of 94 +/- 143 ml (15 +/- 18%, p = 0.001), within the limits of accuracy declared by the manufacturer (20%). The bias was shown to be highly reproducible, either decreasing or increasing the FiO_2 _being 117+/-170 and 70+/-160 ml (p = 0.27), respectively. The EELV measured with the helium dilution method showed a good correlation with the CT scan data (r^2 ^= 0.91) with a negative bias of 136 +/- 133 ml, and appeared to be more correct at low lung volumes.

**Conclusions:**

The EELV measurement with the helium dilution technique (at low volumes) and modified nitrogen washout/washin technique (at all lung volumes) correlates well with CT scanning and may be easily used in clinical practice.

**Trial Registration:**

Current Controlled Trials NCT00405002.

## Introduction

The damage induced by mechanical ventilation in cases of acute lung injury (ALI) or acute respiratory distress syndrome (ARDS) can be termed barotrauma [1], volotrauma [2,3], atelectrauma [4,5] or biotrauma [5,6] depending on the emphasis given to the pathogenic mechanism. They are all caused by the unphysiological stress and strain applied to the whole lung or to particular regions of the ventilated lung [7]. Stress and strain are linked by the specific lung elastance, which has a constant proportionality function [8]. Consequently, knowing the lung stress (i.e. the transpulmonary pressure) or the strain (i.e. the change in volume of the lung relative to its rest position, the functional residual capacity), allows us to better define the mechanical characteristics of the system and to design a safer mechanical ventilation. Although the stress requires the measurement of the oesophageal pressure, the strain requires the measurement of the lung volume.

In the intensive care unit (ICU) setting the measurement of lung volume is not routinely performed; in our practice Ospedale Maggiore Policlinico Mangiagalli Regina Elena the helium dilution technique, which requires an appreciable amount of time and work to become accomplished in, has been used for several years [9,10]. A new technique has been recently proposed (LUFU, acronym for LUng FUnction) based on a modified nitrogen washout/washin technique using a side stream fast oxygen analyser [11]. It is not yet commercially available, but shows a good accuracy in lung volume measurement when compared with the helium dilution technique [12,13].

Another modification of the nitrogen washout/washin technique has been implemented in mechanical ventilation (Engstrom Carestation) [14]. This technique is the subject of the present investigation. The ventilator in which the technique is implemented is equipped with a supplemental pressure port through which the oesophageal pressure could be continuously measured breath by breath. Indeed this ventilator incorporates the technology for measuring both the lung stress (the transpulmonary pressure) and the lung strain (volume change divided by the functional residual capacity) [8,15]. Therefore, to assess the accuracy of the Engstrom Carestation to measure gas volume (and strain) we set up a comparative study. The end expiratory lung volume (EELV), at 5 cmH_2_O airway pressure, was measured by the helium dilution technique, nitrogen washout/washin technique and by computed tomography (CT) scanning, which was taken as the reference gold standard [16].

## Materials and methods

### Study population

Measurements were taken from 30 patients admitted to the ICU from November 2006 to September 2007. The study was approved by the institutional review board of our hospital, and written informed consent was obtained from conscious patients and delayed consent in unconscious patients. Inclusion criteria was the clinical need of a lung CT scan in patients already in mechanical ventilation. Exclusion criteria were age younger than 16 years, pregnancy, haemodynamic instability, documented barotrauma and the presence of chronic lung disease (e.g. chronic obstructive pulmonary disease (COPD)).

### Data collection

The CT scan was performed at end expiration at 5 cmH_2_O positive end expiratory pressure (PEEP), which is the standard pressure at which CT scans are taken for clinical purposes. Thereafter, the patients underwent the measurement of the EELV with the nitrogen washout/washin technique performed with the Engstrom Carestation ventilator and with helium dilution at 5 cmH_2_O PEEP. The nitrogen washout/washin technique was performed by either decreasing or increasing the inspired oxygen fraction (FiO_2_). The average result of these two measurements allowed the accuracy of the Engstrom Carestation in relation to the CT scan to be assessed. Each sequence (decreasing or increasing the FiO_2_) was performed twice to assess the precision (repeatability) of the technique. The accuracy of the helium dilution technique was assessed relative to the CT scan measurement. Only one CT measurement was performed, as the precision of this method has previously been determined [17].

### Quantitative computed tomography analysis

The CT scanner was set as follows: collimation 5 mm; interval 5 mm; bed speed 15 mm per second; voltage 140 kV; and current 240 mA. A whole lung CT scan was performed at a PEEP value of 5 cmH_2_O during an end-expiratory pause. Immediately before each CT scan was obtained, a recruitment manoeuvre was performed. Lungs profile was manually delineated in each cross-sectional lung image in order to identify the regions of interest. Each region of interest was then processed and analysed by a custom-designed software package (Soft-E-Film, University of Milan, Italy), as previously described [18].

We assumed that lung tissue has a density similar to that of water (Hounsefield unit number 0) and considered each voxel to be made of lung tissue and air (Hounsefield unit number -1000). Gas volume can be computed from the Hounsefield number of each voxel according to the following formula:

Gas volume = (CT number/-1000) × voxel volume

The EELV is the sum of gas volumes present in all the voxel included in the lung profiles.

### Nitrogen washout/washin technique

The nitrogen washout/washin technique is based on the following principle: the gas lung volume, at baseline, includes a volume of nitrogen (V_(1)_N_2_) that is determined by the alveolar fraction of nitrogen (F_A_N_2(1)_) (which varies inversely to the alveolar oxygen fraction) and by the EELV accordingly to the following relation:

(1) V_(1)_N_2 _= F_A_N_2(1) _× EELV

If the alveolar nitrogen fraction (F_A_N_2(2)_) is changed by changing the FiO_2_, a new nitrogen volume (V_(2)_N_2_) will be present in the lung after the equilibrium time:

(2) V_(2)_N_2 _= F_A_N_2(2) _× EELV

Assuming that after changing the FiO_2 _the total EELV does not change until the new equilibrium in alveolar gas composition is reached, by subtracting term by term in the equation 1 and 2 the following relation holds true:

(3) VN_2(1) _- VN_2(2) _= (F_A_N_2(2) _- F_A_N_2(1)_) × EELV

as the changes in F_A_N_2 _are specular to the changes in FiO_2_, i.e. ΔF_A_N_2 _= -(FiO_2(1) _- FiO_2(2)_), the EELV can be calculated as:

(4) EELV = ΔN_2 _(ml)/ΔFiO_2_

where ΔN_2 _equals the nitrogen exhaled after the change of inspired FiO_2 _until the equilibration time is reached (about 20 breaths).

The algorithm of the nitrogen washout/washin technique employed by the Engstrom Carestation is detailed by Olegard and colleagues [14]. Nitrogen concentration in expired and inspired air is not directly measured but estimated from the end tidal concentrations of oxygen and carbon dioxide:

(5) ETN_2 _(mmHg) = 713 - ETCO_2 _(mmHg) - ETO_2 _(mmHg)

The alveolar ventilation was calculated as:

(6) Alveolar tidal volume expired = VCO_2_/ETCO_2 _× RR

(7) Alveolar tidal volume inspired = Alveolar tidal volume inspired + ((VCO_2_/RQ + VCO_2_)/RR)

Inspired and expired nitrogen volumes were calculated as:

(8) Expired nitrogen volume = ETN_2_/713 × Alveolar tidal volume expired

(9) Inspired tidal volume = Inspired nitrogen fraction × alveolar tidal volume inspired.

### Simplified helium dilution technique

A flexible tube was inserted between the Y-piece and the patient's endotracheal tube or tracheostomy. The operator clamped the tube during an end-expiratory pause at a PEEP level of 5 cmH_2_O and then connected it to a balloon filled with 1.5 L of a gas mixture of helium (13.07 ± 0.40%) in oxygen. After releasing the clamp, the same operator delivered 10 tidal volumes to the patient in order to dilute the helium gas mixture with the gas contained in the patient's lungs. At the end of this procedure, the balloon was clamped off the circuit, and the patient was reconnected to the ventilator. The concentration of helium in the balloon was then measured by a previously calibrated helium analyser (PK Morgan, Chatham, England). EELV was then calculated using the standard formula: EELV (ml) = (Vb × Ci/Cf) - Vb, where Ci is the helium concentration of the known gas mixture, Cf is the final helium concentration and Vc is the volume of gas in the balloon. The Vb was inflated with 1500 ml of helium-oxygen mixture at 25°C. The volume measured was corrected for body temperature (37°C) using the Gay-Lussac law.

We first performed the CT scan, then the helium dilution technique and at the end the modified nitrogen washout/washin technique. The helium dilution technique and the modified nitrogen washout/washin technique were performed at the CT scan facility without moving the patient from the bed, to maintain the patient's condition. The whole experimental procedure (nitrogen washout/washin technique, helium dilution and CT scan) was performed in a total time of about five minutes.

### Lung mechanics

The total inspiratory resistance of the respiratory system was calculated by dividing the difference in peak inspiratory airway pressure and the plateau inspiratory pressure, measured during an end inspiratory pause, by the inspiratory flow preceding the occlusion. The compliance of the respiratory system was calculated by dividing the plateau inspiratory pressure measured during an end inspiratory pause by the tidal volume.

### Statistical methods

Gas volumes measured with CT scan, nitrogen washout/washin technique and helium dilution technique were compared with the Bland-Altman technique [19] and using a linear regression model. The bias of the EELV measurement performed increasing or decreasing the FiO_2 _were compared using a Student's t-test. Statistical analysis was performed with the R-project software (R foundation for statistical computing, Vienna, Austria [20]).

## Results

Table 1 summarises the clinical characteristics of the patient population. As shown, 66% of the patients could be classified as having ALI with ARDS, 23% could be classified as having ALI without ARDS and 10% had a PaO_2_/FiO_2 _ratio greater than 300.

**Table 1:** The baseline characteristics of the study population. Plateau pressure, respiratory system compliance and arterial blood gas data were measured during the study, standardised at 5 cmH_2_O PEEP.

**Characteristics**	**Value**
Age – years	66 ± 10
Female sex – number (%)	9 (30)
BMI – kg/m^2^	26 ± 8
Tidal volume – ml/kg of predicted body weight	8 ± 1
Minute ventilation – L/minute	7.5 ± 1.8
Respiratory rate – breaths/minute	14 ± 3
Clinical PEEP – cmH_2_O	8.3 ± 3.3
Plateau pressure – cmH_2_O	19 ± 4
Respiratory system compliance – ml/cmH_2_O	43 ± 18
Respiratory system resistance – cmH_2_O/L/second	22 ± 9
PaO_2_/FiO_2 _– mmHg	190 ± 71
FiO_2_	0.50 ± 0.13
PaCO_2 _– mmHg	41 ± 9
Arterial pH	7.422 ± 0.061
***Cause of lung injury – number***	
Pneumonia	6
Sepsis	11
Aspiration	3
Trauma	5
Other	5
***Type of lung injury – number***	
ARDS	20
Acute lung injury	7
Other (PaO_2_/FiO_2 _> 300 mmHg)	3

ARDS = acute respiratory distress syndrome; BMI = body mass index; FiO_2 _= inspired oxygen fraction; PaCO_2 _= partial arterial pressure of carbon dioxide; PEEP = positive end expiratory pressure.

### CT scan and nitrogen washout/washin

**Figure 1:**
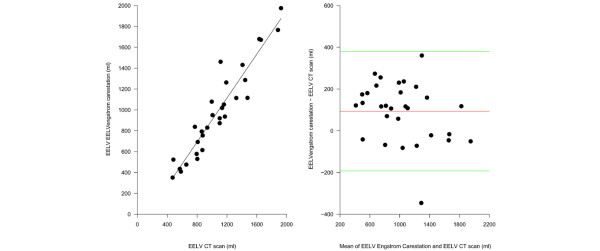
**Comparison of end expiratory lung volume (EELV) measured by the Engstrom Carestation and the computed tomography (CT) scan**. (a)The EELV measured by the Engstrom Carestation as a function of the EELV measured by the computed tomography (CT) scan (EELV carestation = 242 + 0.85 × EELV CT scan, r^2 ^= 0.89, p < 0.00001). (b) The Bland-Altman plot of the EELV measured with the CT scan and the EELV measured with the Engstrom Carestation. The x axis shows the mean of the two measurement and the the y axis shows the difference between the EELV measured by the Engstrom Carestation and the EELV measured by the CT scan (average difference 93 ± 143 ml, limits of agreement -50 – 236 ml).

**Figure 2:**
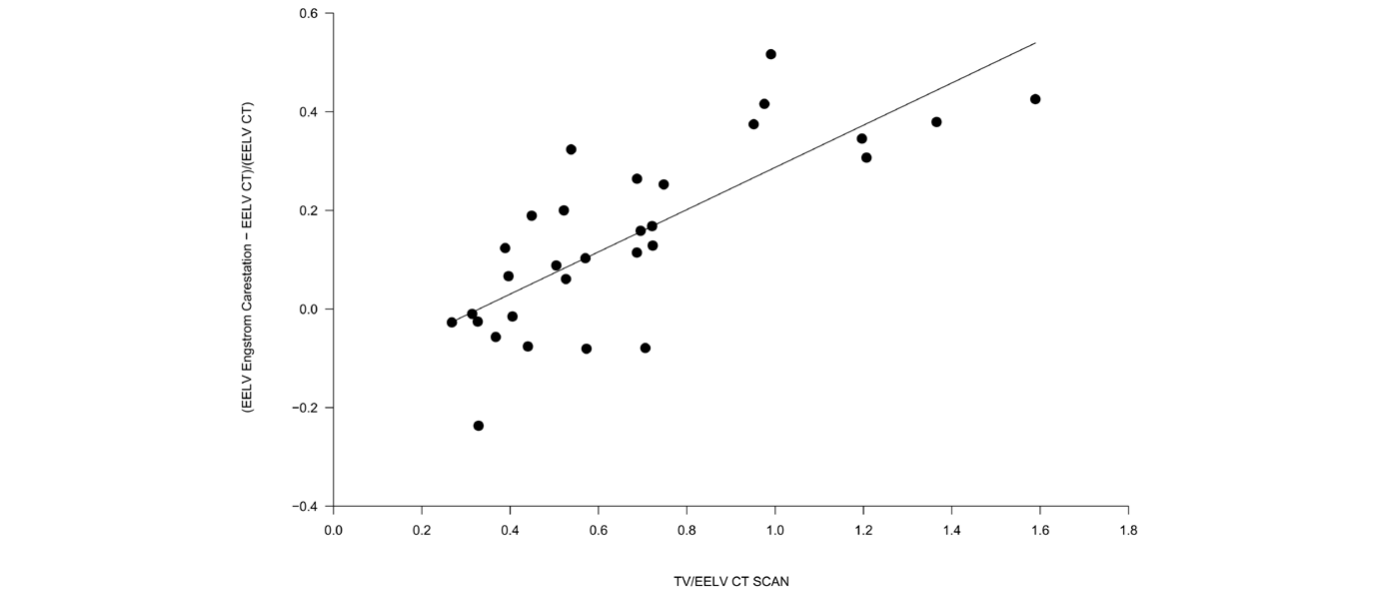
**The relative error of the procedure performed by the Engstrom Carestation**. This is expressed as (EELV_GE _– EELV_CT SCAN_)/EELV_CT SCAN _as a function of the ratio between tidal volume and the end expiratory lung volume (EELV) measured by computed tomography (CT) scan ((EELV_GE _– EELV_CT SCAN_)/EELV_CT SCAN _= 0.05 + 0.43 × (Tidal Volume/EELV_CT SCAN_, r^2 ^= 0.58, p < 0.0001).

As shown in Figure 1a, the EELV measured by the Engstrom Carestation (as the average of two measurements at different FiO_2_), was highly correlated with the EELV computed by the CT scan (r^2 ^= 0.89). The Bland-Altman plot (Figure 1b) revealed a bias of 94 ± 143 ml (15 ± 18%, p < 0.001). The relative error of the simplified nitrogen washout/washin technique (expressed as (EELV_GE _- EELV_CT SCAN_)/EELV_CT SCAN_) was significantly related to the ratio between TV/EELV_CT SCAN _(y = (0.05 + x) × 0.43, r^2 ^= 0.58), as shown in Figure 2.

**Figure 3:**
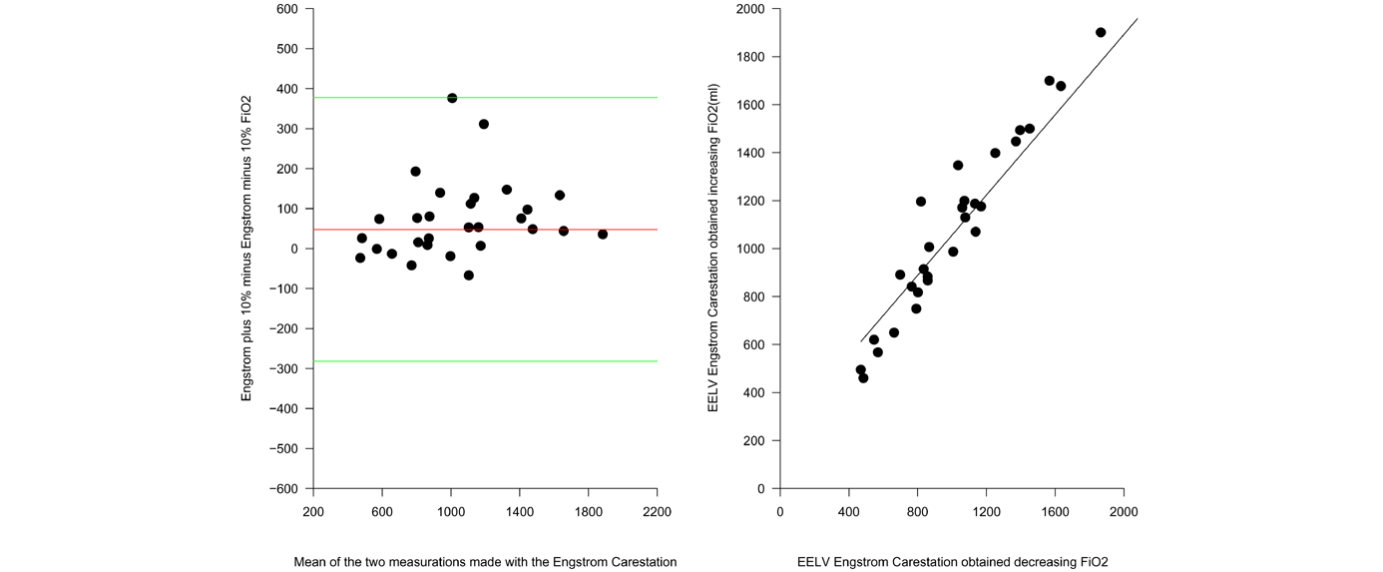
**Accuracy of the nitrogen washin/washout technique**. (a) The relation between the EELV measured by increasing the FiO_2 _as a function of the EELV obtained decreasing FiO_2_. The EELV obtained increasing the FiO_2 _was -56 + 1.0078 multiplied by the EELV obtained decreasing the FiO_2 _(r^2 ^= 0.84, p < 0.0001). (b) The Bland-Altman plot of the EELV measurement obtained increasing the FiO_2_and the EELV obtained decreasing the FiO_2_. The x axis shows the mean of the two measurements and the difference between the EELV measured by increasing FiO_2 _and the y axis shows the EELV obtained decreasing FiO_2 _(average difference 48 ± 165 ml, limits of agreement -117–213 ml).

The accuracy of the nitrogen washout/washin method was similar when either increasing or decreasing the FiO_2_, with a bias of 117 ± 170 and 70 ± 160 (p = 0.27), respectively. The precision of the nitrogen washout/washin measurements is shown in Figure 3 which underlines the high reproducibility of the method with a difference between the two measurements of 48 ± 165 ml, which was not statistically different from zero (p = 0.12).

### CT scan and helium dilution

**Figure 4:**
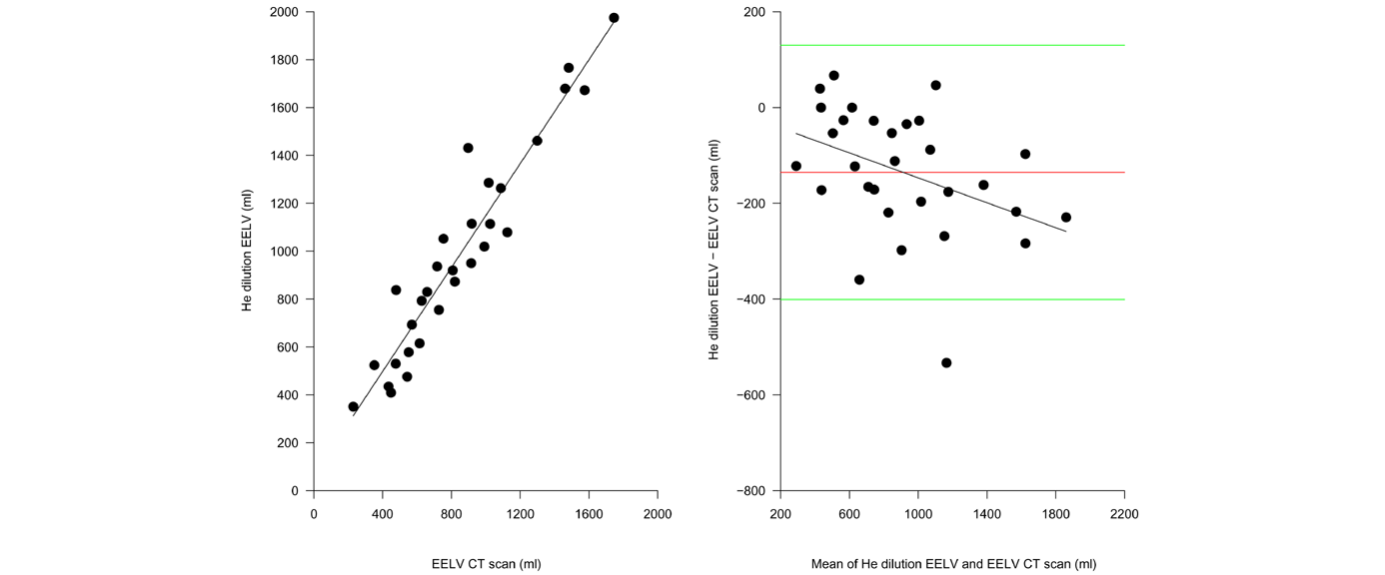
**Comparison of end expiratory lung volume (EELV) measured by the helium dilution technique and the computed tomography (CT) scan**. (a) The EELV measured by the helium dilution technique as a function of the EELV measured by the CT scan (EELV helium dilution = 20 + 0.84 × EELV CT scan, r^2 ^= 0.91, p < 0.00001). (b) The Bland-Altman plot of the EELV measured with the CT scan and the EELV measured with the helium dilution method. The x axis shows the mean of the two measurements and the y axis shows the difference between the EELV measured by the helium dilution method and the EELV measured by the CT scan (average difference -136 ± 133 ml, limits of agreement -3 – 269 ml). The difference between the EELV measured with the helium dilution method and the EELV measured with CT scan was significantly correlated with the EELV, expressed as the average between the two measurements (Helium EELV - CT scan EELV = -15.52764 + -0.17034 × (helium EELV + CT scan EELV)/2, r^2 ^= 0.21, p = 0.005838).

As shown in Figure 4a, the EELV measured by the helium dilution technique was highly correlated (r^2 ^= 0.91) with the EELV computed by the CT scan. The Bland-Altman plot (Figure 4b) revealed a negative bias of -136 ± 133 ml (16 ± 13%, p < 0.001). The Bland-Altman plot also showed a significant negative correlation between the increase of EELV and the difference between the volume measured by the CT scan and the volume measured by the helium dilution technique. Indeed the EELV measured by the helium dilution technique is more accurate at low lung volumes and the accuracy of the measurement decreases when the gas lung volume increases.

### Nitrogen washout/washin and helium dilution techniques

**Figure 5:**
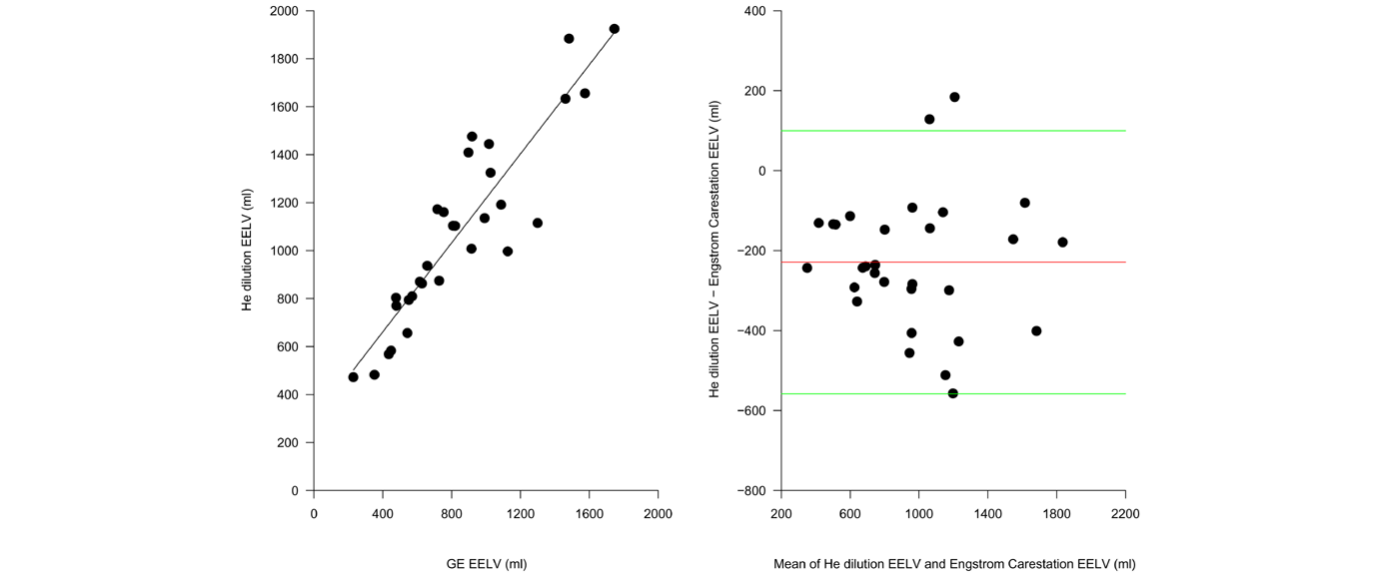
**Comparison of end expiratory lung volume (EELV) measured by the helium dilution technique and the nitrogen washout/washin method**. (a) The EELV measured by the helium dilution as a function of the EELV measured by nitrogen washout/washin method (EELV helium dilution = (290 + 0.92) × EELV GE, r^2 ^= 0.82, p < 0.00001). (b) The Bland-Altman plot of the EELV measured with the nitrogen washout/washin technique and the EELV measured with the helium dilution method. The x axis shows the mean of the two measurements and the y axis shows the difference between the EELV measured by then helium dilution method and the nitrogen washoutwashin measured by the CT scan (average difference -229 ± 164 ml, limits of agreement -558 – 100 ml).

As shown in Figure 5a, the EELV measured by helium dilution technique was well correlated (r^2 ^= 82) with the EELV calculated with the nitrogen washout/washin technique with a negative bias of -229 ± 164 ml (40 ± 26%, p < 0.001; Figure 5b).

## Discussion

The primary finding of this study is that the three different methods we tested (CT scan, simplified nitrogen washout/washin technique and helium dilution technique) to measure the EELV in critically ill patients are in reasonable agreement.

The CT scan measures the lung density and estimates gas volume assuming that the lung is composed of two compartments with very different densities: lung "tissue" and gas [18]. Consequently, CT scans accurately estimate the lung inflation independent of how the different lung regions are ventilated. In contrast, both the nitrogen washout/washin and helium dilution techniques for EELV determination rely on ventilation and measure the fraction of EELV that is ventilable; that is, non-ventilated or poorly ventilated lung compartments will be excluded or underestimated. In this study this fraction is likely to be of minor importance, because we tried to exclude patients with a diagnosis of COPD or other airway disease, in whom the difference between CT measurement and the ventilation-based techniques may be relevant, from our study.

Nitrogen dilution technique was first employed by Durig in 1903 [21] but nitrogen washout/washin techniques did not gain widespread application outside the research setting because nitrogen must be measured with a mass spectrometer. Fretschner and colleagues in 1993 proposed a method to measure the EELV without the need to directly measure the nitrogen concentration [22], but it relied on oxygen and carbon dioxide measurement. The proposed method, however, implied a 30% change in FiO_2 _and the synchronisation between gas measurement and flow measurement. The methodology described by Olegard and colleagues [14] and implemented by the Engstrom Carestation overcomes these two limitations. Synchronisation problems are avoided by calculating the alveolar nitrogen concentration from end-tidal carbon dioxide and oxygen concentrations, and the FiO_2 _changes are limited to only 10%, which can be considered safe even in critically ill patients. In our experimental setting this simplified nitrogen dilution technique showed an average overestimation of lung volume of 94 ± 143 ml (15 ± 18%, p < 0.001), within the limits of accuracy declared by the manufacturer (20%). The technique also proved to be highly reproducible, either by increasing or decreasing the FiO_2_. Indeed it appears to be suitable for clinical application.

In this study we found that the simplified helium dilution technique had a negative bias of 136 ± 133 ml (16 ± 13%) in respect to CT scan; the bias of the helium dilution technique increased linearly with increasing EELV. The simplified helium dilution technique we used, however, was developed to measure the small lung volume of ARDS patients ('baby lung'); it is possible that in patients with a higher EELV the equilibrium in the system (balloon plus lungs) is not reached with 10 normal breaths. The equilibrium time is the function of the time constant of the system (i.e. the time taken to reach approximately 63% of its final (asymptotic) value), which is the product of the compliance of the respiratory system and airway resistances. The time constant is short in ALI/ARDS patients (low compliance), although it is high in patients with COPD, emphysema or any condition that increases lung compliance and airway resistance.

The helium dilution technique differs from the nitrogen washout/washin technique in that no fresh gas ventilation occurs and a reduction in lung volume due to oxygen absortive phenomena is possible, even if data present in the literature show that its effect should be negligible [23,24]. Accordingly the difference between the EELV measured with the CT scan and the EELV measured with the helium dilution method was negligible at lower lung volumes and become consistent increasing the EELV. The helium dilution technique was performed in 10 breaths, while the simplified nitrogen washout/washin technique required 20 breaths to reach equilibrium and this may in part account for the difference between the two techniques based on ventilation. Moreover, the helium dilution method is prone to other sources of error such as the helium concentration in the gas tank, which usually has a tolerance degree of about 5%, and possible diffusion of helium out of the balloon. In addition the possibility of helium uptake during EELV measurement has been discussed in the literature and is thought to be controversial [25-27]. Theoretical considerations show that the possible helium uptake is limited: the solubility coefficient of helium is 1.13 × 10^-5 ^ml/ml/mmHg [28] with a maximal body helium uptake of about 45 ml in 40 L body water and about 5 ml in well perfused body compartments.

## Conclusion

The CT remains the gold standard for measuring gas lung volume. The helium dilution technique is clinically acceptable when applied in patients with a short time constant of the respiratory system. The nitrogen dilution technique appears to be simple and effective.

## Key messages

• The EELV measured with the modified nitrogen washout/washin technique well correlated with the CT scan and is useful for clinical purposes.

• The EELV with helium dilution technique correlated well with the CT scan at low lung volumes but showed a systematic underestimation at greater lung volumes.

## Abbreviations

ALI: acute lung injury; ARDS: acute respiratory distress syndrome; COPD: chronic obstructive pulmonary disease; CT: computed tomography; EELV: end expiratory lung volume; FiO_2_: inspired oxygen fraction; PEEP: positive end expiratory pressure; ICU: intensive care unit.

## Competing interests

LG received lecture fees from GE Healthcare.

## Authors' contributions

DC was responsible for patient selection, clinical conduction of the study and participated in writing the manuscript. MC prepared the database, analysed the data and drafted the manuscript. MC participated in clinical conduction of the study. FT participated in clinical conduction of the study. MB, VB and CM analysed the CT scan data. LG was responsible for study design and participated in writing the manuscript. All the authors read and approved the final manuscript.
